# Some Account of a Tumour, Induced by Hæmorrhage, from Rupture of the Nutritious Artery, within the Femur; with Comments on the Treatment

**Published:** 1835-04-01

**Authors:** John Howship

**Affiliations:** Lecturer on Surgery in the Medical School at the Charing-Cross Hospital; and Surgeon to That Hospital, and to the St. George's Infirmary.


*Some Account of a Tumour, induced by Hæmorrhage, from Rupture of the Nutritious Artery, within the Femur; with Comments on the Treatment.* By John Howship, Esq., Lecturer on Surgery in the Medical School at the Charing-Cross Hospital; and Surgeon to that Hospital, and to the St. George's Infirmary.

The following case may be considered interesting, on either of several accounts; from the close resemblance of its symptoms to those of fungoid disease; from the advantage the patient might perhaps have derived from a more exact view having been taken of the case; or, lastly, from the characters of the disease, which proved a tumour, apparently induced by the rupture of some branch of the medullary artery, within the femur, the artery having continued to bleed during the progress of the disease.

I shall first give the notes of the case, as I collected and set them down the day after the death of the patient.

Sept. 17th, 1832. M. W., aged twenty-three years, about five months since, in descending a staircase, passed down two stairs, instead of one, by mistake; and, as she thought, strained her right knee, on the inner side. She described the injury as confined to a spot exactly beneath the central part of the vastus internus muscle; not at first painful or tender on pressure, but situated, as she said, within the bone. More or less occasional pain, and progressive tumour, were the consequences of the accident; the pain, swelling, and lameness still increasing, notwithstanding the means used for her relief. Her father said that for some time, latterly, she had suffered dreadfully.

By the kind attention of Mr. Anderson, who, towards the conclusion of the case, had seen the patient, I was not only enabled to be present at the examination; but also, with the father's consent, to remove the bone, for more minute subsequent investigation.

The tumour was situated just above the knee, on the inner front of the thigh, the size of a moderately large melon; diminishing as it extended upwards, terminating just above the middle of the femur, and yielding an obscure fluctuation. The leg and foot were loaded with œdema. The integuments, (which were healthy,) when laid aside, exposed the tense fascia, within which a fluctuation was now more distinctly perceived. The fascia, and thin substance of the expanded muscles beneath, being divided, the cyst was laid open, from which a quantity of bloody serum flowed out. The opening being freely enlarged, the contents of the tumour appeared to be traversed by fine membranous septa, whose interstices were filled with grumous blood, masses of fibrine, and serum. In several parts, large coagula of blood were found, confined by a thin film of expanded cellular membrane. The membranous expansions in some parts were rather more firm than in others: at one point was observed, to a trifling extent, a fine lamina of a cartilaginous substance.

The femur, for the extent of the tumour, was exposed: it felt rough, and at the lower part was extensively laid open, by the active operation of progressive absorption, by which the inner condyle was nearly destroyed. Upon the linea aspera, a light and delicate osseous fabric had for some extent been deposited. Several portions of the detached periosteum were recognized in the tumour, involved in the fibrinous blood: the medullary cavity of the bone being filled with coagula. The lower part of the femur was brought away, and laid in water, to macerate.

November 15, 1832. I called upon the father and sister; from whose account I set down the following particulars.

About ten days after the accident, she first felt a little pain, which was compared to rheumatism: at that time she walked as well as ever, and till a month afterwards, when she first began to walk occasionally lame.

About a month after the injury, walking with her sister, she suddenly stopped, and said "Oh dear me; what a pain I have got in the knee! I do not think I can go any further." In a short time this pain went off, and she reached home very well. The part now began to swell a little, and was somewhat tender above the knee, if pressed.

Seven weeks after the accident, she left the family with whom she had lived, and came home; and, in walking to her own house, (a distance of nearly two miles,) said she felt a throbbing pain; but it was trifling. For the following three weeks she could bear, and walk, upon the limb, as firmly as ever. She repeatedly had leeches, blisters, and poultices applied.

On the 18th of June, she went into St. George's Hospital, where she staid about a month, using the means just mentioned, and also applying, as it appeared, an ointment containing tartarized antimony. Urged to submit to amputation, she left the hospital, and returned home.

About six weeks before she died, a distinguished surgeon kindly visited her, at her own house, and assured her that, "if she were the queen of England, and worth the Indies in gold, no medical man could do her any good;" and humanely cautioned her, therefore, not to go to any expense with this view.

At that time the tumour, very large, was always in pain: if, at any moment, she dropped off to sleep for a few minutes, she would jump up suddenly, as if a dart had shot through her thigh. She moaned much in her sleep, and, if awake, was often convulsed, as if in a fit; when her father, perhaps, inquiring "Mary, are you in pain?" she would reply, "Yes, father: I am quite sensible."

She frequently felt severe pains, like pins and needles, passing rapidly upwards, from the toes to the hip, and through to the back; the limb, towards the close of her sufferings, becoming swelled, and oppressed with œdema.

For the last nine or ten weeks, she was not able to stand at all; yet was said to have been but little feverish, to the last; although latterly, she took large and frequent doses of laudanum, with scarcely any effect, her sufferings frequently inducing her to pray earnestly for death.

The femur, cleared from the soft parts, presented the following appearances. The cylinder of the bone was not enlarged, but had lost much of its substance, by progressive or interstitial absorption, which had entirely removed the anterior and internal portions of the inner condyle, exposing the cancellated structure, also extensively destroyed by the same agency; so that, of the internal condyle, the thin articular surface, covered by its cartilage, was nearly all that remained. Neither had the fine vascular membranes lining the longitudinal canals, within the compact substance of the shaft of the bone, been less active; for these canals, extensively and considerably enlarged, had at certain points become united laterally with each other, inducing a loss of strength to the solid bone, with numerous openings upon its external surface.

Upon the linea aspera, new bone had to some extent been deposited; but on no other part of the femur.

An attentive examination of this preparation demonstrates, that absorption had commenced within the medullary cavity; the internal parts having suffered most materially, the substance of the cylinder less, and the outer surface least; proving that the cause of the disturbance and destruction of the osseous fabric must have been seated within the cancellated cavity. Even in those parts where the cancellous structure yet remains, in the seat of the disease, its condition is entirely changed; for, if observed with a good magnifying glass, it will be perceived that, instead of a reticulated texture, of rounded fibres, the whole is reduced, apparently from pressure, to so many exquisitely fine, thin, flattened plates, here and there entirely disappearing, in consequence of absorption having completed their removal.

From the above recent and ultimate appearances of this disease, illustrated as they are by the previous symptoms, there seems no reason to doubt that the whole was the progressive effect of continued pressure and excitement, disturbing the healthy functions, first within the medullary cavity, and subsequently among the soft parts beneath the fascia of the thigh, consequent to the rupture of some branch of the nutritive artery of the bone; and that the case may therefore be regarded as an instance of progressive internal hæmorrhage.

That a small artery, under particular circumstances, may continue to pour out its blood for a great length of time, is sufficiently demonstrated by an interesting specimen in my collection, in which a ruptured vessel did continue to bleed, inducing the slowly increasing effects of compression upon the cervical portion of the spinal cord, and occupying more than twelve months before it destroyed life;* and the tumour in the thigh, in the present case, was blood, and blood only. Some masses were apparently recent coagula, others partially changed, the fibrine having separated itself from the serum, and at certain points, to some extent from the colouring matter; yet the yellow tinge, and peculiar tenacious elasticity of this substance, could not, even in this state, be readily mistaken.

From the commencement and progress of the symptoms in the above case, it might well have been supposed a case of fungoid disease, and it may fairly be presumed that this was the opinion of those gentlemen who saw it, during its progress, judging from the advice given her; and this certainly was my own impression on first observing the tumour, after death. The dissection, however, set this matter right; and subsequent reflection suggesting a doubt as to whether any, or what, operation ought to have been performed, brought to my recollection several cases, but one particularly, in which I had found all the soft parts of the thigh inundated by fluid and coagulated blood, in an extensive diffused aneurism, the consequence of a wound in the femoral artery, which, when the cavity had been laid open and cleared, and the artery tied, did exceedingly well; the soft parts relieving themselves

* The history is published; being the 30th Case, in my Practical Observations in Surgery and Morbid Anatomy.

with ease from their temporary embarrassment. Again, from what I heard of the patient, M. W., and from what I have seen of the powers and disposition of healthy bone in other cases, my opinion is that, if even in the advancing stage of the disease, she had been advised to undergo an operation, the object of which was to save, not to remove, the limb, it would in all probability have been submitted to; and that, had the tumour then been laid freely open, turning out the coagula, and, if hæmorrhage had rendered it necessary, applying a cautery to the cancellated structure, (provided the cavity had been dressed with lint, the limb fomented, and the system soothed,) healthy suppuration and granulation would have followed, and the young woman, in all probability, would have perfectly recovered.

Even the extensive loss of bone sustained at the time of her death, would not, in my opinion, have precluded the possibility, or perhaps have lessened even the probability, of recovery. There is no reason to doubt that the bone would have granulated, or that the periosteum would, very soon, have resumed its healthy functions; the probable result, in a constitution otherwise healthy, being an abundant deposit of new bone within the medullary cavity, or upon the surface of the weakened cylinder, or in both these situations, enabling the limb to support the weight of the body, eventually, as well as ever.

Neither would the operation for opening the tumour have been unadvisable, in any event; for, had the disease proved to be fungus hæmatodes, the making a section through the tumour in the first instance would not have prejudiced or impeded the next required step, the removal of the limb. The doubt also previously explained, the patient's mind would have been prepared for the uncertainty of the event, according to what the disease might have been found under examination.

That new and compact bone would have been secreted, had the proposed operation been performed, may be inferred on better evidence than mere opinion: it may be proved, by the following example, in which not more, but less, might have been expected from the constitutional powers than in the case now under consideration; an example in which the disease was spina ventosa, with much expansion of the bone; while in M. W.'s case, there was interstitial absorption only, from the pressure and irritation of effused blood, inducing no expansion at all.

March 16, 1829. My friend Mr. Spilsbury sent A. W., a young woman, from Walsall, for my opinion concerning a tumour situated two thirds down the inner side of the left tibia. The swelling, first observed the size of a pea, had been four years in acquiring its present dimensions, about three inches in length, two in breadth, and half an inch in elevation; attended with occasional shooting or throbbing pain.

The tumour felt equable, rather heated, and as if supported by a light ossific shell, except at one point, where Sir ASTLEY COOPER (whose opinion was requested on the case,) detected a deep-seated fluid. We both agreed that the tumour contained a fluid, and that it was not a case of fungoid disease.

April 23. Mr. SPILSBURY had the goodness to bring me the osseous shell, forming the anterior part of the tumour, which he had removed a few days before by an operation. The cavity was found of considerable size, filled with a bloody serum; the medullary arteries (several nearly the size of crow-quills,) bled profusely, but the hæmorrhage was restrained by the prompt and free application of the actual cautery, assisted subsequently by compression. We called with the specimen on Sir A. COOPER, who observed that Mr. GREEN and himself had lately had a patient on whom a similar operation was performed: the patient went on well for some months, and the wound healed, but the disease returned; and he feared that would also be the result in the present case.

The thin osseous shell, removed by the saw, was externally even, but internally divided into various cells, some of which still contained a grumous fibrine.

June 17, 1831. I received a letter from Mr. SPILSBURY, stating that, although by the former operation, the progress of the disease had been so far arrested, that the wound had granulated, and healed to the size of a split pea, repeated escharotic applications (at the suggestion of other practitioners,) had induced irritation, and the limb had been eventually amputated, in the Birmingham Hospital. At the same time I was favoured with the bone itself, the section of which demonstrates, in a very interesting manner, the extent to which nature had carried forward the work of reparation. So far, indeed, as to have left scarcely a trace of the cellular character of the original tumour; the cancellated cavity being occupied with an ossific deposit, of healthy appearance, far exceeding in compactness of texture the ordinary cancellated structure. A curious circumstance also, as remarked by Mr. S., was, that the surrounding marginal line of the tumour had, since the operation, become much diminished in breadth, the tibia having consequently recovered much of its natural figure.

Having now stated the case, the appearances on dissection, and the remarks to which those appearances gave rise; it only remains to add, that, desirous to learn if any mode of relief had been proposed in such cases, or whether many other cases, precisely resembling it, had been observed, I turned to a valuable memoir on this subject, referred to by my friend Mr. Benjamin Bell, in his new work on the Diseases of the Bones. It is a memoir, by M. Breschet, extremely well drawn up, copiously illustrated by cases, full of practical interest, and leading to this conclusion, that, as such tumours bears strong traces of analogy to aneurism in soft parts, the most appropriate treatment is that adopted in aneurism, by the ligature of the principal artery of the limb. The most important case adduced, perhaps, is one in which this treatment was adopted, and that, as will presently be seen, with very decided advantage; although the disease was not permanently cured. A brief outline of this case I shall take the liberty to transcribe, with certain conclusions the author has laid down, regarding the treatment; and I regret that circumstances, which prevented my devoting more time, prevented also my looking more fully into the merits of so instructive a communication.

"C. N. Renard complained of a tumour, on the external part of the right tibia, just below the knee-joint, beating with the pulse. In about a year, the limb lost the power of support, and bent under him.

"He was taken into the Hôtel Dieu, in 1819. The tumour was of the size of the palm of the hand, with occasional shooting pains. The beating ceased, on compressing the femoral artery. M. Dupuytren thought it a dilatation of the arterial capillaries of the part; and, local applications failing to relieve, he determined to take up the femoral artery. The operation performed, at once arrested the pulsation in the tumour. He did well; and at the time of quitting the hospital, only a slight tumefaction remained.

"In 1826 he again entered the Hôtel Dieu, with a return of the tumour to a much greater extent than ever. The superficial veins were exceedingly enlarged, but no pulsation was now perceived in the tumour, which was thirty-two inches in circumference. The limb was amputated, and the patient recovered.

"The tumour of the amputated limb was of enormous volume; a development of the upper extremity of the tibia. On the front, one or two soft but tense projecting points had now become sunk, and flaccid, where the ossific shell was deficient.

"On dissection, the popliteal artery was of its natural size. The articular arteries, not enlarged, but very small, when injected with care, were seen to send their small filaments into the tumour behind. The recurrent branch of the anterior tibial artery, on the contrary, much enlarged, corresponded to the supply of the swelling on the anterior part of the tibia; its numerous branches expanding over, and penetrating into the substance of the bone.

"The venous system was so much enlarged, that the internal saphena vein was the size of the little finger. The tibia was enormously increased in size, and its natural form at the upper part entirely lost. Divided longitudinally, the cavity of the tumour was exposed. The space was distributed into compartments and cells; one of the anterior and largest contained gelatin, the cavity being invested with a highly vascular membrane. Other cells contained a similar secretion, and some a yellow or black and putrescent matter. The vascular network of some cells was injected; the cavities of others filled with layers of albumen, formed by coagulated blood, as in aneurism."

After detailing a considerable number of extremely interesting cases, M. Breschet observes that

"If it is proved beyond a doubt that this change in the osseous tissue arises from a disease in the arteries of the medulla, and that they are enlarged, it is natural to suppose that the ligature of the trunk supplying these vessels would be the best, and only treatment that could be adopted; a conclusion favored not only by the analogy of all other cases of aneurismal disease, but by the effect of the ligature in Renard's case, the success of which demonstrates the analogy of these diseases of the ossific tissues with other forms of aneurism.

"In aneurism of the arteries of soft parts, we may previously try local applications, or compression of the tumour, but in the present case we can hope nothing from these expedients. The ligature, then, is the only means in which we can trust for success.

"Admitting that fungoid tumours in soft parts, and those called fungoid tumour of the periosteum, are identical with the disease just described in the osseous tissue (which M. Breschet does not allow) the frequent communication of arteries from different trunks leads to an opinion that the ligature of the principal artery would not be likely to succeed, or that its influence would be confined to lessening the present volume of the tumour, checking its progress for some time; although the circulation re-establishing itself by the numerous vascular communications, the disease would soon return to the state it was in before the operation.

"For the same reason, we see the swelling and pulsation so often return, in tumours of erectile tissue, after tying the principal artery; but in the bones the arterial circulation does not possess the same facilities for re-establishing itself, when the trunk furnishing the principal nutritious arteries has been tied; consequently, this operation offers the best mode of treating this disease in bone.

"Again, is the ligature advisable in every case? Experience shews that the chance of success is greater, the earlier the operation is performed. We may remark, that where it has not succeeded in curing the disease in the osseous tissue, the peculiar characters of aneurism have not returned, and the tumour has remained stationary a very long time.

"If in Renard's case, organic disease, many years after the use of the ligature, required amputation of the limb: it was the consequence of the accidental circumstance of the vessel being secured at a period when not only disease of the vascular tissue existed, but also an organic change in the condition of the bone, very distinct from that of the vessels. What happened here in the osseous fabric, occurs also in the soft parts, after the operation for aneurism when the operation has been too long deferred.

"It is in the present day well known that this operation is the most successful, when, instead of trifling, the ligature is applied in the early period of the disease. Mr. HODGSON has perfectly established this important truth, in his work; and contrary to all previously received opinions, has incontestably proved that all delay in applying the ligature in aneurism, lessens the probability of success.

"If we have sufficiently demonstrated the advantage and indispensable necessity of the ligature in aneurism of the arteries of the osseous tissue, we think also that amputation is the only resource when the change in the state of the bone is far advanced, and attended with deep-seated disease in the medullary and cancellated structure.*"

Now, it is evident that the case of Renard, as related by M. BRESCHET, was by no means an exact parallel to that of M. W. It much more nearly resembled that which occurred under the care of Mr. SPILSBURY; in the tumour being connected with expansion of the bone, in my mind an essential distinction, and a character of which there was no trace in the instance at the head of the present paper.

While therefore the taking up the principal artery of the limb might probably have been the most appropriate treatment in the cases brought forward by M. BRESCHET, most of which seem to answer the description of spina ventosa, and especially the case of Renard, in which the advantage of this treatment was demonstrated; it appears to me that in the case of M. W. the symptoms might have been relieved, and the disease probably cured, with much less eventual inconvenience to the limb, than is frequently incurred by an operation, the very object of which is permanently to set aside the direct course of the circulation, through the limb.

It is true that where the operation for popliteal aneurism has been required in early life, when the elasticity of the arterial system has afforded every facility to success, very little ultimate inconvenience has been the result. But I have in several cases, in middle-aged persons, observed that the perfectly free transmission of the blood through the limb is never afterwards restored; in one instance, at present under my eye, in which a man recovered from that disease, by the

* Observations et Reflexions sur les Tumeurs Sanguines, &c. Par G. Breschet. (Repertoire Générale d'Anatomie et de Physiologie Pathologique. tom. 2.)

natural cure taking place, the inconvenience subsequently felt has been such, as almost completely to deprive him of the power of taking exercise at all. This man, entirely easy, and able to move about with freedom a short distance, is yet, after a few minutes' walking, invariably so overpowered by cramps, violent and intolerable pain, as to be obliged to stop short, and stand still for half an hour, till the limb has recovered itself.

In conclusion, then, it may perhaps be doubtful whether, in cases like those above related, connected with expansion of the bone, the removal of a part of the tumour, as in Mr. Spilsbury's case, or the taking up the femoral artery, as in M. Breschet's case, be the most advisable course. The reason I have just given would even here induce me rather to prefer the operation adopted by Mr. S., especially if a seton were subsequently inserted, or an issue kept open, for one or two years, near the seat of the disease.

But, on the other hand, should the placing a ligature on the principal artery, in such cases, be regarded the better expedient (and I am fully aware that either this or any other suggestion should have great weight, from so distinguished an authority as M. Breschet,) then I would say, let the cases be justly discriminated; let those that manifest an enlargement of the bone, and an osseous shell, be submitted to the ligature; but let those deep-seated tumours which, as in the instance brought forward in the present paper, betray, under examination, no indication of expansion of the bone, or the presence of a bony case, be first subjected to the section I have advised, and, if necessary, to the operation I have ventured to advocate, previously to the limb being condemned to amputation.

---

